# Performance, Egg Quality, and Yolk Antioxidant Capacity of the Laying Quail in Response to Dietary Choline Levels

**DOI:** 10.3390/ani12233361

**Published:** 2022-11-30

**Authors:** Osman Olgun, Esra Tuğçe Gül, Gözde Kılınç, Alpönder Yıldız, Abdullah Çolak, Ainhoa Sarmiento-García

**Affiliations:** 1 Department of Animal Science, Faculty of Agriculture, Selçuk University, 42130 Konya, Turkey; 2 Department of Food Processing, Suluova Vocational Schools, Amasya University, 05500 Amasya, Turkey; 3 Research and Application Farm, Faculty of Agriculture, Ankara University, 06110 Ankara, Turkey; 4 Área de Producción Animal, Department of Construcción y Agronomía, Facultad de Ciencias Agrarias y Ambientales, Universidad de Salamanca, 37007 Salamanca, Spain

**Keywords:** antioxidant capacity, choline, egg quality, performance, quail

## Abstract

**Simple Summary:**

Adequate dietary nutrient intake is essential for optimal performance in poultry production. In recent years, improvements in animal genetics have led to changes in animal nutrition requirements. Consequently, livestock nutrient requirements must be periodically reevaluated. Choline is an essential nutrient for poultry and its deficiency causes developmental and health problems. Choline requirements for laying quail are based on previous recommendations and require new research. This study aimed to determine the ideal dose of choline for laying quails based on their productive development and egg quality. A dose of 1500 mg/kg of choline was determined to be sufficient to maintain egg quality and performance of laying quails. Nevertheless, 3500 mg/kg of choline would be required in the diet to observe improvements in egg antioxidant capacity.

**Abstract:**

The current study determined the ideal dose of choline in the diet of laying quails based on egg development, egg quality, and antioxidant capacity. A total of 120 female quails (244.7 ± 10.38 g) were randomly assigned to 6 experimental groups with 5 replicates of 4 quails each. In the 10-week trial, treatment diets were formed by adding 6 choline chloride−60% concentrations providing 1500 (control), 2000, 2500, 3000, 3500, and 4000 mg/kg of choline. The feed intake of quails was quadratically affected (*p* < 0.05) by the choline level of the diet, in other developmental parameters, and by egg quality among these treatments. The feed intake was reduced to 2500 and 3000 mg/kg levels of choline in the diet compared to the control group. Free radical scavenging capacity (DPPH) of the yolk increased linearly (*p* < 0.001) with dietary choline levels. In contrast, the thiobarbituric acid reactive substances (TBARS) value decreased as dietary choline levels increased, except for 4000 mg/kg levels. Based on the findings of the current study, 1500 mg/kg of dietary choline is adequate to maintain performance parameters and egg quality in laying quails. However, to increase egg antioxidant capacity, in terms of the DPPH value, the dietary choline dose needs to be increased to 3500 mg/kg.

## 1. Introduction

The nutrition of avian species plays a key role in their health, egg quality, and body growth [1,2]. In poultry production, adequate nutrient intake is crucial for optimal performance [1]. An excess or deficiency of nutrients has negative effects on bird health and productivity [3]. There is evidence that genetic improvements to increase animal production can alter nutrient requirements, so it is important to reassess nutrient needs periodically.

Choline is a water-soluble micronutrient, which plays important physiological roles including synthesizing cell membranes and forming acetylcholine. It functions as a key component in neurotransmission, membrane integrity, and methylation pathways [1,4,5]. In addition to serving as a methyl donor of choline, it also contributes to liver lipid metabolism, nerve impulses, and cell wall structure [6]. Moreover, choline plays a crucial role in the synthesis of betalipoproteins and phospholipids. Fats can be transported and burned with it, which also prevents fatty liver disease [7].

In day-old chicks, choline is classified as an essential micronutrient because it provides the body with a labile methyl group so that creatine and methionine can be formed. It is essential for bird growth and to prevent perosis [8]. However, there is no agreement on the appropriate dose. There are differences between the results of previous experiments that evaluated the administration of choline in poultry diets on performance. According to some reports [9], the addition of choline to the diets of layer poultry could improve performance parameters, while previous authors [10] proposed that choline supplementation did not affect performance. A similar situation exists for egg quality, which is the second most important marketing factor after performance [11]. There is research proposing that choline supplementation would not change egg quality [12], while other authors considered that it could improve it [13].

In recent years, studies on the functions of choline in poultry have mainly focused on its antioxidant capacity [14,15]. Choline administration decreases liver oxidative stress and improves the overall antioxidant capacity of poultry [13,14,15]. Furthermore, choline appears to be beneficial for laying hens with fatty liver hemorrhagic syndrome and for reducing the risk of fatty liver. This is because choline suppresses abnormal lipid deposition by increasing the intake of fatty acids or by activating their transport as lecithin in the liver [8]. This specific function can influence the composition of meat and eggs related to the lipid profile. Nevertheless, according to Dong et al. [15], there is little literature regarding the effects of choline on lipid profiles other than on total liver lipids, which is very limited.

There is scarcely any research on the choline requirements of laying quails. The lack of information on nutritional requirements can cause food costs to rise, as well as underestimating or overestimating their nutritional needs, resulting in economic losses [11] Choline requirements for laying quails are established at 1500 mg/kg based on National Research Council [16] recommendation. Recent studies proposed that quails can get enough choline from their diet (1546 mg/kg) without supplementation and it would not adversely affect their performance [17]. This could be beneficial, as it would reduce the cost of the feed. However, there is no clear consensus among researchers to determine the appropriate dose of choline for laying quails. Ameen et al. [9] demonstrated that choline levels in the diet of 1800−1950 mg/kg increased both egg production and egg mass. On the other hand, Griep Junior et al. [18] reported that the 1260 mg/kg level of choline should be added to the diet of quails for maximum egg production and egg weight.

The number of studies examining choline requirements or the effect of dietary choline levels on the performance of laying quail is limited. In addition, to our knowledge, there are no studies examining the effect of choline on the antioxidant capacity of the egg yolk. Considering the above information, this study aims to reevaluate choline requirements based on the performance and egg quality of laying quails. This study sought to establish the influence of choline on yolk antioxidant capacity in terms of TBARS and yolk DPPH values.

## 2. Materials and Methods

### 2.1. Ethical Approval

Criteria specified by the European policy for protecting animals [19] were followed during the experimental period.

### 2.2. Animals and Feed Materials

The current experiment was conducted on 120 female quails (*Coturnix coturnix Japonica*) with similar body weight (244.70 ± 10.38 g) and 22 weeks of age at the Department of Animal Science, Faculty of Agriculture, Selcuk University, Turkey (38°1′36″, 32°30′45″). Quails were obtained from a commercial company. The trial lasted 70 days.

A completely randomized design was used for this experiment. Animals were randomly allocated to 5 identical cages that had the same environmental conditions. The study was conducted in 6 experimental groups consisting of 5 replicates, each containing 4 female quails.

Quails were housed in clean and disinfected battery cages (30 cm wide 45 cm long). Quails were maintained in a well-ventilated room with a lighting program of 16 h. A temperature of 20 ± 2.0 °C and a relative humidity of 55 ± 5% were arranged in each pen. Each pen was provided with an individual feeder and drinker to allow ad libitum intake.

The basal diet prepared in mash form was formulated according to the NRC [13] to supply layer quails’ requirements (Table 1). A total of 6 trial diets used in the experiment were formed to contain 1500, 2000, 2500, 3000, 3500, and 4000 mg/kg choline chloride −60% (Feedvit Chem. Technol., Konya, Turkey). The chemical composition of the basal diet was analyzed according to AOAC [20] proceedings.

### 2.3. Determination of Performance Parameters

At the beginning of the experiment, the quails were randomly allocated to the six trial groups. A precision weighing balance (±0.01 g) was used to weigh the quails at the beginning and the end of the experiment. Each group in the trial was weighed initially and at the end of the trial (g) to determine body weight change.

Experimental diets were given by weighing each subgroup, and, subsequently, feed intake was calculated as the daily feed intake per quail. At the same time each day (10:00 a.m.), eggs were collected and recorded. Egg production was determined by dividing the number of eggs obtained in a day by the number of quails and multiplying by 100 and given as a percentage (%).

Egg weight was determined by weighing one by one all of the eggs collected in the last three days of the experiment with a precision weighing balance (±0.01 g). From these data, egg mass was calculated as daily egg weight per quail according to the following Equation (1), as Gül et al. [21] showed in their research.

(1) $$\mathrm{Egg}\;\mathrm{mass}=\frac{\mathrm{egg}\;\mathrm{production}\;\left(\%\right)\times \mathrm{egg}\;\mathrm{weight}\;\left(g\right)\;}{100}$$

Finally, feed conversion ratio was determined according to following Equation (2):

(2) $$\mathrm{Feed}\;\mathrm{conversion}\;\mathrm{ratio}=\frac{\mathrm{feed}\;\mathrm{intake}\;}{\mathrm{egg}\;\mathrm{mass}\;}$$

### 2.4. Determination of Egg Quality Parameters

The number of broken, cracked, and damaged eggs were calculated as a percentage of the total number of eggs used in the experiment. Egg internal and external quality parameters were determined at room temperature at Selcuk University, Faculty of Agriculture, Egg Quality Laboratory from all eggs collected in the last three days of the trial. Eggshell breaking strength was assessed by applying supported-systematic pressure to the blunt of the eggs (Egg Force Reader, Orka Food Technology, Herzliya, Israel).

A clean, glass surface was used to break the eggs immediately after the eggshell breaking strength was determined. The shells were dried at room temperature for three days and then weighed, and relative weights were calculated as a rate of the egg weight (%). Eggshell thickness was calculated by averaging the measurements obtained from three sections (equator, blunt, and pointed parts) of the eggshell using a micrometer (Mitutoyo, 0.01 mm, Kanagawa, Japan). Eggs, for which external quality characteristics were determined, were broken on a surface, their albumen and yolk heights were measured with a height gauge, and their length and width were measured with a 0.01 mm digital caliper.

Based on these data, the following equations were used to calculate the parameters:

Albumen index was determined using the next Equation (3):

(3) $$\mathrm{Albumen}\;\mathrm{index}=\frac{\mathrm{albumen}\;\mathrm{height}\;}{\frac{\mathrm{albumen}\;\mathrm{width}+\mathrm{albumen}\;\mathrm{length}}{2}}\times 100$$

To determinate yolk index, the following Equation (4) was used:

(4) $$\mathrm{Yolk}\;\mathrm{index}=\frac{\mathrm{yolk}\;\mathrm{height}}{\mathrm{yolk}\;\mathrm{diameter}}\times 100$$

Finally, the Haugh unit (5) for each egg was calculated using data of egg weight and albumen height according to the equation proposed by Haugh [22]:

(5) $$\mathrm{Haugh}\;\mathrm{unit}=100\times \log\left(\mathrm{albumen}\;\mathrm{height}+7.57-1.7\times {\;\mathrm{EW}}^{0.37}\right)$$

For colorimetric analysis, samples were deposited on the flat surface of Petri dishes, and all analyses were performed to maintain the integrity of the egg yolks. According to Titcomb et al. [23], egg yolks were subjected to a previously calibrated Konica Minolta digital colorimeter (Minolta Chroma Meter CR 400 (Minolta Co., Osaka, Japan) for the measurement of the parameters L* (lightness), a* (redness), and b* (yellowness).

### 2.5. Determination of TBARS and DPPH Levels of Yolks

A thiobarbituric acid reactive substances (TBARS) assay was performed as a modified method described by Kilic and Richards [24] and Sarmiento-Garcia et al. [25] in triplicate from each group to determine lipid peroxidation. A two-gram sample was taken from the yolk and added to 12 mL of the trichloroacetic acid (TCA) solution (7.5% TCA, 0.1% EDTA, 0.1% Propyl gallate). Then, the mixture was homogenized in an ultraturrax (IKA, Wilmington, NC, USA) for 20 s and filtered through Whatman nr 1 filter paper (Maidstone, England). Following this, the solution was placed in glass tubes (3 mL), and 3 mL of the thiobarbituric acid (TBA) solution (0.02 M) was added and vortexed. The mixture was heated for 40 min in a boiling water bath (100 °C) to develop a pink color. After cooling, the sample was centrifuged at 2000 rpm for 5 min. The supernatant was measured spectrophotometrically at 530 nm wavelength using a spectrophotometer (Perkin Elmer, Waltham, MA, USA) against a blank containing 1 mL TCA extraction solution and 1 mL TBA solution. The TBARS were calculated using a standard curve of malondialdehyde, the decomposition product of tetraethoxypropane (TEP), which was used for the preparation of the standard curve. TBA value (6) was calculated as µmol MDA/g yolk using the following equation, as Kilic and Richards [24] proposed:

(6) $$\mathrm{TBA}\;\mathrm{Value}=\frac{\left(\mathrm{absorbance}/k\;\times 2/1000\right)\times 0.8\;}{\mathrm{sample}\;\mathrm{weight}}\times 100$$

The antioxidant activity of obtained hydrolysates was assessed based on the radical scavenging effect 1, 1-diphenyl-2-picrylhydrazyl (DPPH)-free radical activity according to the modified method of Sacchetti et al. [26]. A two-gram sample was taken from the yolk and was added to 25 mL of 95% methanol (100%), and extraction was performed in falcon tubes in an ultrasonic bath for 20 min. The mixture was filtered through Whatman nr 1 filter paper (Maidstone, England) and sampled into 0.1 mL glass tubes. A volume of 2.9 mL of DPPH solution (100 mL of methanol (100%)) + 0.0025 g of DPPH (97%) was added to the mixture and mixed in a vortex for 25 s. Then, the mixture was left for 30 min at room temperature, and the absorbance of the solution was measured at 517 nm wavelength using a spectrophotometer (Perkin Elmer precisely UV/VIS Spectrometer, Waltham, MA, USA). The control was operated in the same way, with 95% ethanol used to replace the sample solution. Each experiment was repeated 3 times to determine the average value. DPPH values were calculated according to the following Equation (7), proposed by Sacchetti et al. [26]:

(7) $$\mathrm{DPPH}\;\mathrm{values}=\frac{[\left(\mathrm{control}\;\mathrm{absorbance}-\mathrm{sample}\;\mathrm{absorbance}\right)}{\mathrm{control}\;\mathrm{absorbance}}\times 100$$

### 2.6. Statistical Analysis

Data were analyzed by one-way ANOVA using the SPSS 22.0 software package (SPSS Inc., Chicago, IL, USA), using the cage means as an experimental unit. A probability value of *p* < 0.05 was considered statistically significant. Orthogonal polynomial contrasts were used to assess the significance of linear and quadratic models to describe the response of the dependent variable to a rising dietary choline level.

## 3. Results

All quails were alive at the end of the trial, and neither mortality nor illness symptoms were observed among the groups. According to the data in Table 2, the administration of choline to the diet at different levels did not statistically affect performance parameters (in terms of final body weight, body weight change, egg production, egg weight, egg mass, and feed conversion ratio). However, feed intake was quadratically affected by dietary choline levels (*p* = 0.001). The feed intake of the groups fed with diets containing 2500 and 3000 mg/kg choline (32.70 and 32.58 g/day/quail, respectively) was lower than in the levels of 1500 and 4000 mg/kg (34.57 and 34.31 g/day/quail, respectively), while the feed intake values of the rest of the groups were similar. Moreover, a trend (*p* = 0.052) was observed for the feed conversion ratio. Based on the results, it appears that as the concentration of choline in the diet increases, the feed conversion ratio decreases.

The effects of the dietary supplementation of choline on the egg’s external quality are given in Table 3. The data presented that eggshell breaking strength (1.60 kg), eggshell weight (8.51%), and eggshell thickness (241.9 µm) were higher at a dietary choline level of 2000 mg/kg than at other choline levels. However, those differences were not significant (*p* > 0.05). Moreover, the level of dietary choline did not affect the damaged egg rate (*p* > 0.05).

As shown in Table 4, the minimum and maximum values of egg internal quality parameters were found as follows: albumen index (2.15−2.83); yolk index (42.17−43.70); Haugh unit (84.41−88.96); L* value of yolk (50.97−52.24); a* value of yolk (0.178-1.078); and b* value of yolk (31.79−35.70). However, dietary choline intake did not affect any of the internal egg quality parameters. (*p* > 0.05).

According to Table 5, yolk DPPH and TBARS concentrations were affected by the addition of choline to the diet linearly and quadratically, respectively (*p* < 0.001). Regarding yolk TBARS levels, they were significantly higher (*p* < 0.001) in the groups that had received 2500, 3000, and 3500 mg/kg choline in the diet than in the rest of the experimental groups. Moreover, free radical scavenging capacity was determined as DPPH reduction to evaluate antioxidant activity spectrophotometrically. The yolk DPPH value was higher (*p* < 0.001) in the quails that received 3500 and 4000 mg/kg choline than in 1500 and 2000 mg/kg, while the rest showed intermediate values. The increase in dietary choline from 1500 mg/kg to 4000 mg/kg resulted in a 17.45% increase in yolk DPPH levels.

## 4. Discussion

Choline plays several important physiological roles, including synthesizing cell membranes and forming acetylcholine, and is a necessary ingredient in poultry nutrition. So, to maintain animal health and prevent diseases, it is important to include an adequate level of choline [27]. In the current research, feed intake of the quails fed with a diet containing 2500 and 3000 mg/kg choline decreased (*p* = 0.001) compared to the control group (1500 mg/kg). In addition, increasing choline levels also showed a trend (*p* = 0.052) of reducing the feed conversion ratio. The results of the current experiment partially agree with the findings of Jahanian et al. [28], who found a decrease in feed intake and improved feed conversion ratio values when choline (1000 mg/kg) was added to broiler diets. Similarly, Alagawany et al. [29] reported a decrease in feed intake when choline was included in the diet at levels above 2500 mg/kg. These authors proposed that choline supplementation would increase metabolic rate, and consequently these parameters would be higher, which is consistent with the results obtained in the current research.

The rest of the performance parameters were not affected by the dietary choline level. The effect of choline supplementation on the performance parameters of laying quails and hens does not seem to be clear. The current findings are consistent with previous authors who stated that the choline level did not affect the performance parameters of laying hens [30,31], growing quails [7,17], and broilers [32]. This evidence supports the result of this study regarding production performance, which showed no difference. According to Gregg et al. [32], the high basal concentrations of choline in the diet may have impacted the ability to determine meaningful differences in growth performance. Moreover, these authors proposed that methionine and choline have connected biosynthetic pathways. The lack of response to increasing additions of choline chloride could be explained by the presence of methionine in the diet. These authors proposed that the improvement in development would be more evident when methionine requirements are deficient. In the current study, no differences were observed between dietary treatments for egg production, egg weight, or egg mass. These parameters are highly interrelated. An increase in egg mass is usually correlated with an increase in egg production, since egg mass is the result of egg production multiplied by egg weight [12]. The absence of differences in egg production parameters could presumably be due to the same supply of nutrients, such as protein, energy, and fat, present in the feed. Variations in the energy, protein, and fat content of the feed could affect egg production. Conversely, Griep Junior et al. [18] reported that the administration of choline at levels up to 1260 mg/kg to laying quails improved feed efficiency without changing feed intake or egg production. Khairani et al. [12] revealed that the addition of 1500 mg/kg choline to low methionine quail diets had a positive impact to enhance egg production and egg weight, but had no effect on feed intake. The higher egg weight in quails fed low methionine diets supplemented with choline chloride suggests an important relationship between choline and methionine, which both contribute methyl groups to egg synthesis. Methionine’s methyl donation can be replaced by choline, and the methyl of choline can be used for methionine synthesis. Similarly, Ameen et al. [9] stated that choline supplementation of 1800 or 1950 mg/kg in the diet was shown to be effective at improving egg production, but did not affect feed intake. It is possible that the discrepancy between the previous results and those shown in this study may be due to the dose of choline, the period of supplementation, the composition of the basal diets, the dietary fat level, the sex, or the genotype of the subjects [28].

Regarding egg external quality parameters, no differences were found between experimental diets. The eggshell thickness had the same relative value for all experimental groups because the treatment diets had the same Ca and P contents. Several factors affect the eggshell thickness, including minerals such as calcium, magnesium, and phosphorus, which are major inorganic components [12]. Consequently, the eggshell breaking strength and the percentage of damaged eggs did not differ among the different experimental groups. These findings are in line with the observations of Khairani et al. [12], who clarified that the choline level (1498 or 2998 mg/kg) in the diet did not change egg quality in quails. In other studies, dietary choline level was found to affect at least one egg quality parameter. Yonke and Cherian [13] identified that the supplementation of choline (2460 mg/kg) to layer hen diets improved egg quality. Another researcher [31] expressed that the supplementation of choline at levels up to 1260 mg/kg to laying hens did not affect egg quality, except for eggshell breaking strength. Griep Junior et al. [18] reported that the maximum eggshell weight in quails was obtained with the addition of 840 mg/kg choline to the diet, but this effect was not observed for the Haugh unit. Zhai et al. [33] found that the choline level of the diet improved albumen and yolk color score. Choline is a major component in the formation of the phospholipid lecithin, a component of egg yolk. Contradictorily, this study showed that choline supplementation did not affect the yolk index, Haugh unit, or albumen index. In all cases, the methionine requirements were met, which can explain this fact. An increase in these parameters is evident when methionine levels are deficient [32].

Choline is effective at improving the antioxidant capacity of the liver [14,15] and preserving the oxidative stability of meat [28] and eggs [34]. In the current study, the DPPH level of the yolk increased along with the dietary choline level, especially at 3500 and 4000 mg/kg levels. Therefore, the linear increases in DPPH indicated that dietary choline could increase the antioxidant capacity of eggs. According to Moghadam [34], the intramolecular hydroxy amine group of choline functions as a decomposer of hydroperoxides by donating an electron, which contributes to reducing lipid peroxidation and protein oxidation in choline-supplemented groups. This response to choline supplementation could result from its role as a methyl donor, which can indirectly increase antioxidant capacity [35,36]. Methionine plays an important role in the synthesis of adenosylmethionine and GSH, which are two important antioxidants [37]. The addition of choline, which is a methyl donor, to the diet has a reducing effect on the use of methionine [12]. Probably, the improved yolk oxidative stability observed in the present study could also be explained by choline levels and/or its metabolites’ accumulation into the egg yolk, and the reduction in the rate of lipid peroxidation caused in the lipid fraction of the yolk. In addition, choline donates a methyl group to tetrahydrofolate, which reduces large quantities of NADPH that can be used to regenerate glutathione, and ultimately vitamin E, which stabilizes and reduces the availability of lipids for peroxidation [37]. For the reasons mentioned above, the addition of choline to the diet can enhance the effect of these nutrients as antioxidants by having a protective effect on methionine and vitamin E. Likely, these effects could also be observed in the meat of these birds. To our knowledge, there are no studies examining the effect of choline on yolk DPPH concentration in poultry. On the other hand, in a study conducted on pigs, it was reported that the addition of choline to the diet did not change the DPPH level of meat [35]. Nevertheless, these authors found that the other parameters studied that evaluate scavenging activities (O_2_, H_2_O_2_) were improved with the inclusion of choline in obese pigs.

Lipid peroxidation, one of the most important factors affecting eggshell life, is determined by TBARS [36]. As a result of this study, choline at a concentration of 1500 and 2500 mg/kg resulted in the lowest TBARS value (*p* < 0.05), while above these levels TBARS value was negatively affected, except for at the 4000 mg/kg choline level. These results are in partial agreement with those shown by Dong et al. [15]. This study indicates that choline (up to 6800 mg/kg) may adversely affect lipid profiles and redox status in layer diets, and yolk phosphatidylcholines and total yolk lipids respond more sensitively to dietary choline supplementation than other indicators. Conversely, the current results contradicted the findings of Yonke and Cherian [13] because they reported that dietary choline levels did not significantly affect the TBARS value of the yolk. On the other hand, Moghadam et al. [34] stated that choline supplementation (1500 mg/kg) to hen diets containing flaxseed decreased the TBARS level of the yolk. These differences could be attributed to the age of the animals and the duration of the experiment. Current results demonstrate that choline improves DPPH levels of the yolk, but the effect on the yolk TBARS level is unclear. Therefore, further studies are needed to understand these findings.

## 5. Conclusions

In conclusion, a diet containing 1500 mg/kg of choline is sufficient for quail laying performance and maintaining the quality of their eggs according to NRC recommendations [16]. However, to improve the antioxidant capacity of the egg, in terms of DPPH value, it would be necessary to include doses of 3500 mg/kg of choline in the diet of laying quails. Further studies are needed on the effect of dietary choline levels on feed intake, antioxidant properties, and especially lipid oxidation.

## Figures and Tables

**Table 1:** Basal diet and its nutrient content.

Ingredients	g/kg	Nutrient Content	g/kg
Yellow corn	570.0	Metabolizable energy (kcal/kg)	2898.41
Soybean meal (46% CP)	261.0	Crude protein	200.42
Full-fat soybean	65.0	Crude fiber	28.30
Meat-bone meal (45% CP)	27.6	Crude fat	53.28
Sunflower oil	17.1	Moisture	126.29
Limestone	52.0	Lysine	9.94
Salt	3.0	Methionine	4.49
Premix ^1^	2.5	Cystine	4.10
DL-methionine	1.8	Calcium	25.04
Total	1000.0	Total phosphorus	6.18
		Available phosphorus	3.50
		Choline (mg/kg)	1476

CP: Crude Protein. ^1^ Premix (vitamin-mineral mixture) as contained per kg: Vitamin A, 8000 IU; vitamin D^3^, 3000 IU; vitamin E, 5 mg; vitamin K, 2 mg; vitamin B_12_, 0.02 mg; biotin, 0.1 mg; folic acid, 1 mg; niacin, 50 mg; pantothenic acid, 15 mg; pyridoxine, 4 mg; riboflavin, 10 mg; thiamin, 3 mg; copper, 10 mg; iodine, 1.0 mg; iron, 50 mg; manganese, 60 mg; zinc, 60 mg; selenium, 0.42 mg incorporated at 1 g/kg of feed.

**Table 2:** Effect of dietary choline levels on performance of laying quails.

Parameters		Dietary Choline Levels (mg/kg)					S.E.M.*	*p*-Values		
	1500	2000	2500	3000	3500	4000		Anova	L	Q
**Body weight change (g)**	27.3	24.0	38.4	18.6	39.9	33.1	7.08	0.290	0.361	0.881
Final body weight (g)	269.4	271.7	282.0	263.6	288.2	274.8	8.55	0.430	0.439	0.794
Egg production (%)	91.69	91.86	94.39	91.43	91.45	94.29	1.348	0.457	0.474	0.806
Egg weight (g)	12.29	12.34	12.51	12.40	13.01	12.77	0.368	0.753	0.199	0.936
Egg mass (g/quail/day)	11.25	11.34	11.79	11.33	11.90	12.05	0.358	0.119	0.119	0.829
Feed intake (g/quail/day)	34.57 ^a^	33.91 ^ab^	32.70 ^b^	32.58 ^b^	33.12 ^ab^	34.31 ^a^	0.932	0.029	0.362	0.001
Feed conversion ratio (g feed/g egg)	3.07	3.01	2.78	2.88	2.79	2.87	0.088	0.194	0.052	0.152

S.E.M.*: Standard error means; L: Linear effect; Q: Quadratic effect; ^a,b^: Means with different superscripts in the same row were significantly different (*p* < 0.05).

**Table 3:** Effect of dietary choline levels in laying quail diets on egg external quality parameters.

Parameters		Dietary Choline Levels (mg/kg)					S.E.M.*	*p*-Value		
	1500	2000	2500	3000	3500	4000		Anova	L	Q
**Damaged egg (%)**	0.00	0.33	0.56	0.71	0.00	0.58	0.337	0.763	0.568	0.530
Eggshell breaking strength (kg)	1.42	1.60	1.52	1.58	1.39	1.57	0.081	0.431	0.883	0.593
Eggshell weight (%)	8.33	8.51	8.27	8.35	7.77	8.35	0.255	0.468	0.361	0.804
Eggshell thickness (µm)	235.4	241.9	232.7	239.8	235.2	241.3	3.760	0.521	0.540	0.589

S.E.M.*: Standard error means. L: Linear effect; Q: Quadratic effect.

**Table 4:** Effect of dietary choline levels in laying quail diets on egg internal quality parameters.

Parameters		Dietary Choline Levels (mg/kg)					S.E.M.*	*p*-Value		
	1500	2000	2500	3000	3500	4000		Anova	L	Q
**Albumen index**	2.78	2.69	2.48	2.83	2.15	2.70	0.170	0.121	0.277	0.448
Yolk index	43.70	42.91	42.84	42.72	42.17	42.70	1.062	0.954	0.421	0.638
Haugh unit	88.25	88.52	86.41	88.79	84.41	88.96	1.420	0.287	0.627	0.397
L*	51.30	51.28	52.24	50.97	51.22	51.22	0.835	0.925	0.793	0.728
a*	0.178	0.863	0.751	1.078	0.925	0.765	0.4622	0.834	0.400	0.328
b*	33.14	33.07	35.70	31.85	33.31	31.79	2.648	0.301	0.355	0.308

L*: Lightness; a*: Redness; b*: Yellowness. S.E.M.*: Standard error means; L: Linear effect; Q: Quadratic effect.

**Table 5:** Effect of dietary choline levels on DPPH and TBARS values of yolks in laying quails.

Parameters		Dietary Choline Levels (mg/kg)					S.E.M.*	*p*-Value		
	1500	2000	2500	3000	3500	4000		Anova	L	Q
**TBARS (µmol MDA/g yolk)**	3.78 ^b^	3.75 ^b^	4.30 ^a^	4.49 ^a^	4.31 ^a^	3.59 ^b^	0.155	<0.001	0.495	<0.001
DPPH (%)	5.56 ^b^	5.62 ^b^	5.97 ^ab^	6.05 ^ab^	6.30 ^a^	6.53 ^a^	0.195	0.009	<0.001	0.794

S.E.M.*: Standard error means; L: Linear effect; Q: Quadratic effect; DPPH: 2,2 diphenyl-1-picrylhydrazyl; TBARS: Thiobarbituric acid reactive substances; MDA: Malondialdehyde; ^a,b^: Means with different superscripts in the same row were significantly different (*p* < 0.05).

## Data Availability

The data analyzed for the study are available from the corresponding author upon reasonable request.
